# Quantitative Assessment of Polarization and Elastic Properties of Endometrial Tissue for Precancer/Cancer Diagnostics Using Multimodal Optical Coherence Tomography

**DOI:** 10.3390/diagnostics14192131

**Published:** 2024-09-25

**Authors:** Anton A. Plekhanov, Gennady O. Grechkanev, Elena A. Avetisyan, Maria M. Loginova, Elena B. Kiseleva, Anastasia A. Shepeleva, Alexander A. Moiseev, Alexander A. Sovetsky, Ekaterina V. Gubarkova, Anastasia A. Anina, Angelina M. Shutova, Sergey V. Gamayunov, Grigory V. Gelikonov, Vladimir Y. Zaitsev, Marina A. Sirotkina, Natalia D. Gladkova

**Affiliations:** 1 Privolzhsky Research Medical University, 10/1 Minin and Pozharsky Sq., 603950 Nizhny Novgorod, Russia; ggrechkanev@mail.ru (G.O.G.); marialoginova96@yandex.ru (M.M.L.); kiseleva84@gmail.com (E.B.K.); kgybarkova@mail.ru (E.V.G.); aninaan419@gmail.com (A.A.A.); flamingo.go1@yandex.ru (A.M.S.); sirotkina_m@mail.ru (M.A.S.); natalia.gladkova@gmail.com (N.D.G.); 2 Nizhny Novgorod Regional Oncological Hospital, 11/1 Delovaya St., 603093 Nizhny Novgorod, Russia; elenamedvedeva77@yahoo.com (E.A.A.); nastya20.murom@gmail.com (A.A.S.); gamajnovs@mail.ru (S.V.G.); 3 A.V. Gaponov-Grekhov Institute of Applied Physics The Russian Academy of Sciences, 46 Ulyanova St., 603950 Nizhny Novgorod, Russia; aleksandr.moiseev@gmail.com (A.A.M.); alex.sovetsky@mail.ru (A.A.S.); grgel@yahoo.com (G.V.G.); vyuzai@mail.ru (V.Y.Z.); 4 Lobachevsky University, 23 Gagarin Av., 603022 Nizhny Novgorod, Russia

**Keywords:** endometrial hyperplasia, cancer, optical coherence tomography, cross-polarization OCT, elastography, attenuation coefficient

## Abstract

**Objectives**: The most important phase in the endometrial pathologies diagnostics is the histological examination of tissue biopsies obtained under visual hysteroscopic control. However, the unclear visual diagnostics characteristics of subtle focal endometrial pathologies often lead to selection errors regarding suspicious endometrial lesions and to a subsequent false pathological diagnosis/underestimation of precancer or early-stage cancer. **Methods**: In this study, we investigate the potential of Multimodal Optical Coherence Tomography (MM OCT) to verify suspicious endometrial lesion regions before biopsy collection. We study the polarization (by cross-polarization OCT, CP OCT) and elastic (by compression OCT-elastography, C-OCE) properties of ex vivo endometrial tissue samples in normal conditions (proliferative and secretory phases to the menstrual cycle, atrophic endometrium) with endometrial hyperplasia (non-atypical and endometrial intraepithelial neoplasia) and endometrial cancer subtypes (low-grade, high-grade, clear cell and serous). **Results**: To the best of our knowledge, this is the first quantitative assessment of relevant OCT parameters (depth-resolved attenuation coefficient in co-[Att(co) values] and cross-[(Att(cross) values] polarizations and Young’s elastic modulus [stiffness values]) for the selection of the most objective criteria to identify the clinically significant endometrial pathologies: endometrial intraepithelial neoplasia and endometrial cancer. The study demonstrates the possibility of detecting endometrial pathologies and establishing optimal threshold values of MM OCT criteria for the identification of endometrial cancer using CP OCT (by Att(co) values = 3.69 mm^−1^, Sensitivity (Se) = 86.1%, Specificity (Sp) = 92.6%; by Att(cross) values = 2.27 mm^−1^, Se = 86.8%, Sp = 87.0%) and C-OCE (by stiffness values = 122 kPa, Se = 93.2%, Sp = 91.1%). The study also differentiates endometrial intraepithelial neoplasia from non-atypical endometrial hyperplasia and normal endometrium using C-OCE (by stiffness values = 95 kPa, Se = 87.2%, Sp = 90.1%). **Conclusions**: The results are indicative of the efficacy and potential of clinical implementation of in vivo hysteroscopic-like MM OCT in the diagnosis of endometrial pathologies.

## 1. Introduction

Endometrial (endometrioid) cancer (EC) is the second most common female gynecologic malignancy with a steadily increasing incidence [1]. There is growing evidence that the diagnosis of EC may be associated with the prior development of endometrial hyperplasia [2,3,4], which, among other things, share common risk factors for the disease [5]. However, even though some atypical forms of endometrial hyperplasia are direct precursor lesions of EC, the incidence of endometrial hyperplasia is approximately three times greater than EC [6]. Therefore, the latest clinical practice guidelines emphasize the need not only to diagnose EC at an early stage but also to identify among endometrial hyperplasia the cases with the endometrial intraepithelial neoplasia (EIN) as a precancer lesion with significant malignant potential [7,8,9]. Nevertheless, the valid diagnosis and detection of endometrial pathologies is a difficult challenge due to the specific characteristics of the studied object. In particular, an endometrial pathology is a dynamic multicellular tissue structure that undergoes hormonally induced cyclical changes in its morphological structure [10]. In addition, EIN/EC are pathomorphological heterogeneous processes with multifaceted architectural and cytological features, appearing as focal or diffuse lesions, the diagnosis of which is often associated with the underestimation of pathologies [11,12].

The main stage of diagnosing endometrial pathologies is the histological examination of tissue taken from the uterine cavity [13]. For many decades, the most common methods of obtaining endometrial tissue for subsequent histological examination were highly traumatic Pipelle biopsy and dilation and curettage [14,15]. In addition to high trauma, which is unwanted in young women who desire to preserve future fertility [16], such methods of blind sampling demonstrate low specificity in diagnosing focal endometrial pathologies (which is often observed in EIN/early-stage EC) [17,18]. For these reasons, hysteroscopy-guided biopsy is currently considered the gold standard in EIN/EC diagnostics. This allows for visualization of the uterine cavity and focal lesions, from which it is possible to take tissue biopsy [19] or even sometimes completely remove the lesion (hysteroscopic resection) under visual control [20]. Hysteroscopy-guided endometrial biopsy has also been shown to have a higher diagnostic accuracy than dilation and curettage [21]. Common visual hysteroscopic parameters indicating the presence of EIN/EC are local or diffuse thickening of the endometrium with a papillary or polypoid appearance, abnormal vascular pattern, the presence of glandular cysts and glandular outlets demonstrating abnormal architectural features [22]. In EC, gross distortions of the endometrial cavity are most often visualized due to the nodular, polypoid, papillary or mixed nature of tumor growth [23]. Focal necrosis, microcalcifications and loose tissue consistency may also be observed, allowing for a fairly high accuracy of detection at a late-stage EC. However, the approximate visual characteristics for the diagnosis of subtle focal lesions (such as EIN/early-stage EC) often lead to errors in identifying the tissue region of endometrial lesions for hysteroscopy-guided biopsy [24,25]. This is also associated with the moderate values of the diagnostic parameter of hysteroscopy sensitivity in EIN diagnosis, which is limited to ~75% [26]. Also, hysteroscopy is beneficial in visually localizing a suspicious lesion, but it does not provide any information about its pathomorphology and tissue structure at the histology level. In these cases, the incorrect selection of a suspicious endometrial area for subsequent histological examination often leads to a misleading pathological diagnosis of endometrial hyperplasia and an underestimation of EC [27]. Therefore, a search for a new diagnostic tool for targeted, non-traumatic, high-resolution examination of the endometrial morphological structure to identify endometrial tissue lesions with EIN/EC is merited and highly desirable.

At the current level of diagnostic technologies development, the optical coherence tomography (OCT) seems to be an appropriate method for the above-mentioned tasks [28]. The prospects for the gynecological use of OCT were described in [29], and the possibility of in vivo non-traumatic intrauterine OCT imaging of human endometrial tissue has been demonstrated by the authors earlier [30]. In this paper, we for the first time demonstrate the capabilities of multimodal (MM) OCT for the diagnostics of human endometrial pathologies. The multimodality of OCT technology represents the possibility of simultaneously obtaining intelligence on the cross-polarization and elastic properties of the studied bio-tissue, which could lead to the establishment of the most optimal criteria for the detection and differentiation of endometrial pathologies. One way or another, as estimated by MM OCT (with high-resolution ~10 µm), the bio-tissue’s properties represent its morphological architecture, which certainly undergoes significant changes in its development toward malignancy. There are a few recent studies demonstrating the high potential of using classical OCT cross-sectional images (B-scans) to differentiate endometrial hyperplasia or EC from the normal endometrium [31,32]. In contrast to the signal intensity assessment, our study calculates the depth-resolved attenuation coefficient [33], which allows us to objectify and unify the obtained results independently of the used OCT system. In addition, we for the first time study the cross-polarization (CP) and elastic properties of human endometrial tissue. The CP modality is a variant of polarization-sensitive OCT that allows for estimation of the initial polarization state changes due to both birefringence and cross-scattering in bio-tissues [34]. It has been shown that CP OCT increases the efficacy of diagnostics of pathologies accompanied by changes in the connective tissue component (for example, in the diagnosis of bladder cancer [35,36] or breast cancer [37,38]). The elastic properties of bio-tissue were assessed by compression optical coherence elastography (C-OCE), which relies on estimation of the gradient of signal phase variation during the uniaxial compression of studied tissue by the optical surface of the OCT probe [39]. It has been shown that C-OCE allow studying the morphological structure of bio-tissue based on the difference in the elastic properties of morphological components and pathomorphological changes (for example, when diagnosing breast cancer [40,41], prostate cancer [42,43] or colorectal cancer [44,45]). With regard to endometrial tissues, we assumed the simultaneous use of different OCT modalities can allow the identification of the most effective MM OCT criterion or their combinations for the diagnosis of endometrial pathologies. Thus, this paper describes detailed MM OCT studies of ex vivo surgical samples of endometrial tissue that are normal (from premenopausal and postmenopausal women), with endometrial precancer (non-atypical hyperplasia and EIN) and cancerous (different morphological subtypes). The aim of this work is to establish the most objective and effective MM OCT criteria for the identification and differentiation of EIN/EC. Establishing such criteria will determine the possibilities and feasibility of using OCT as a clinical tool for non-traumatic high-resolution diagnostics of clinically important endometrial lesions for the subsequent implementation of targeted tissue biopsy in order to reduce the incidence of underestimation of precancer/cancer.

## 2. Materials and Methods

### 2.1. Patients and Experiment Design

The study adhered to the international and ethical standards of the Helsinki declaration of the World Medical Association “Ethical principles for medical research involving human subjects” [46]. The study was approved by the Institutional Review Boards of the Nizhny Novgorod Regional Oncological Hospital (protocol#14 of 7 April 2022). All patients included in the study provided written informed consent prior to enrollment.

The study included 57 patients (premenopausal and postmenopausal women 33–73 years) treated in the Nizhny Novgorod Regional Oncological Hospital between 2023 and 2024. The diagnosis was made based on the results of histological evaluation post-hysteroscopy-guided biopsy/dilation and curettage of the uterus. All patients underwent total hysterectomy (surgical procedure that removes uterus). The following groups of patients were studied and graded by pathologic classification according to the current clinical guidelines [7,8,47]: (i) without pathological changes in the endometrium, undergoing hysterectomy for indications not related to uterine pathology, including premenopausal women in the proliferative (*n* = 3) and secretory (*n* = 4) phases of the menstrual cycle, and postmenopausal women with an atrophic endometrium (*n* = 11); (ii) with hyperplastic changes in the endometrium, including non-atypical endometrial hyperplasia (*n* = 8) and atypical hyperplasia/EIN (*n* = 3); with various pathological subtypes of EC—low-grade (G1 and G2) EC (*n* = 13), high-grade (G3) EC (*n* = 6), clear cell EC (*n* = 5) and serous EC (*n* = 4).

Immediately after the completion of surgery, one fresh tissue sample per patient (~2.0 cm × 1.0 cm × full thickness of the uterine wall) was obtained. The samples were delivered to the laboratory in 10% bovine serum albumin solution on ice within 10–15 min of the resection. MM OCT imaging of each tissue sample included a sequential obtaining of CP OCT and C-OCE data. The study was performed from the surface of the endometrium and took ~30 min (time varies from the sample size). Immediately after the MM OCT imaging, the samples were subjected to histological examination.

### 2.2. Multimodal Optical Coherence Tomography (MM OCT)

A spectral-domain MM OCT device “OCT 1300-E” (BioMedTech Inc., Nizhny Novgorod, Russia) was used to assess uterine samples. The setup combines traditional and cross-polarization structural OCT (CP OCT) [34] and C-OCE [48] imaging. This MM OCT system has a common-path interferometric layout and utilizes a source with a central wavelength of 1.3 µm, a spectral width of 100 nm and a receiving array with a 20 kHz rate of A-scan acquisition [49]. This device enables axial resolution of 10 µm, lateral resolution of 15 µm, and scanning depth of ~1.7 mm in air. During one signal-acquisition cycle, the OCT system generates a 3D data set 256 pixels in depth (~1.7 mm in air) and 512 × 512 pixels laterally (2.4 × 2.4 mm) consisting of 512 B-scans (cross-sectional OCT images) in 26 s. Only the central cross-sectional B-scan corresponding to C-OCE and histological images was subjected to subsequent analysis. The OCT system is equipped with a flexible fiberoptic probe, which is designed for a contact examination of bio-tissue. For spatial positioning of the OCT-probe and implementation of controlled compression during C-OCE imaging, we used a 3D positioning-compressing system PLRA4 (Purelogic R&D Inc., Voronezh, Russia) [41,44]. It performs 3D positioning of the OCT probe with an accuracy of 10 µm. The use of this setup enabled both high-precision lateral positioning and vertical motion of the probe required for performing controlled compression of the studied samples during C-OCE examinations.

Structural CP OCT imaging includes the simultaneous recording of two images from one tissue area. They are an image in the co-polarization (reflected light with a polarization state parallel to the initial polarization state) channel and an image in the cross-polarization (reflected light with changed polarization, which is orthogonal to the initial one) channel [34]. To enhance contrast and enable quantitative assessment, CP OCT images were converted to attenuation coefficient maps. To calculate the attenuation coefficient, a depth-resolved method was used [50] with some modification to compensate for additive noise and the sensitivity of the OCT device (which was to avoid any systematic error in estimating the attenuation coefficient) [33]. The distribution of attenuation coefficient values for each OCT images is presented as rainbow color-coded maps in a cross-section plane, coinciding with the histological images plane. The minimum and maximum values of the color scale (blue and red, respectively) were selected taking into account the optimal contrast (the resulting value was within 0–12 mm^−1^).

C-OCE imaging enables the assessment of elastic (stiffness) properties of bio-tissues. In C-OCE imaging, the compression-produced strains were estimated by the elastographic processing of the acquired phase-sensitive OCT scans [48]. Quantification of the tissue stiffness was achieved by comparing the strain in the tissue and in the reference pre-calibrated layer of linearly elastic translucent silicone placed on the sample surface [51]. During the processing, interframe axial strains in the visualized area were calculated by estimating the axial gradient of interframe phase variations using the ‘vector’ method [52] supplemented by an additional optimization of the processing parameters [53]. Furthermore, using the so-obtained interframe strains, cumulative strains were calculated simultaneously in the reference silicone and underlying tissue to obtain stress–strain dependences for the tissue [54]. Since the stress–strain dependences of bio-tissues often are nonlinear, for reproducible quantification of the tissue stiffness values (or Young’s elastic modulus), the tangent Young’s modulus was estimated for a pre-chosen level of applied stress: 1 kPa [55]. The so-obtained C-OCE images were represented as color-coded maps, such that stiffer areas are shown in blue, and softer areas are shown in red (stiffness values scale bar was within 0–600 kPa).

Further quantitative analysis of attenuation coefficient maps/C-OCE images included the random selection of 10 regions (10 × 10 speckles) located in endometrium layer projection and calculation of the average values of the attenuation coefficients in the co- (Att(co)) and cross- (Att(cross)) polarization channels/the Young’s elastic modulus (Stiffness) for each region. The total amount of analyzed values included statistics of ~100–500 measurements depending on the number of examined samples for each group of endometrial morphology.

### 2.3. Histological Examination

After MM OCT imaging of the freshly excised sample, the area of scanning was marked with histological ink. The sample was then fixed in 10% formalin for 48 h and cut through the marked area to prepare the histological sections coinciding with the plane of co-, cross-polarization OCT and C-OCE images. For the histological examination, hematoxylin and eosin staining was used. The sections were imaged using All-in-one Type EVOS M7000 Imaging System (Thermo Fisher Scientific Inc., Waltham, MA, USA).

Histological sections of uterine tissue samples were assessed by a pathologist, and all 57 cases were confirmed consistent with a preoperative hysteroscopy-guided biopsy/dilation and curettage of the uterus. In order to outline the areas of endometrial tissue with norm and pathologies in the histology images, the QuPath software v0.5.1 (QuPath, Edinburgh, UK) for morphometrical analysis was used [56].

### 2.4. Statistical Analysis

The Att(co), Att(cross) (mm^−1^) and Stiffness (kPa) values were used for statistical inter-group comparison. Descriptive statistics results are expressed as Me [Q1; Q3], where Me is the median value of stiffness; Q1, Q3 represent are the 25th and 75th percentile values, respectively. As this study involves a comparison of several groups of morphological structures/pathologies of the endometrium, the Mann–Whitney U-test with Bonferroni correction was chosen. In all cases, differences were considered statistically significant when *p* < 0.05.

The assessment of the informative value and diagnostic capabilities of the studied MM OCT criteria was carried out with an estimation of Sensitivity (Se) and Specificity (Sp). Based on the Se and Sp values, the receiver operating characteristic (ROC) curves were constructed, which show the dependence of the true positive rate on the false positive rate [57]. The higher the AUC is, the better the classifier. Statistical analysis was performed on GraphPad Prism 8.0 (GraphPad Software, La Jolla, CA, USA).

## 3. Results and Discussion

Results are presented as follows and discussed in two subsections: (i) a qualitative and quantitative analysis of the obtained MM OCT data set from the study of endometrial tissue in normal and pathological conditions, and (ii) calculation of diagnostic parameters and establishment of the most objective MM OCT criteria for the diagnosis and differentiation of EIN/EC.

### 3.1. MM OCT Analysis of Polarization and Elastic Properties of Endometrial Tissue

**Figure 1:**
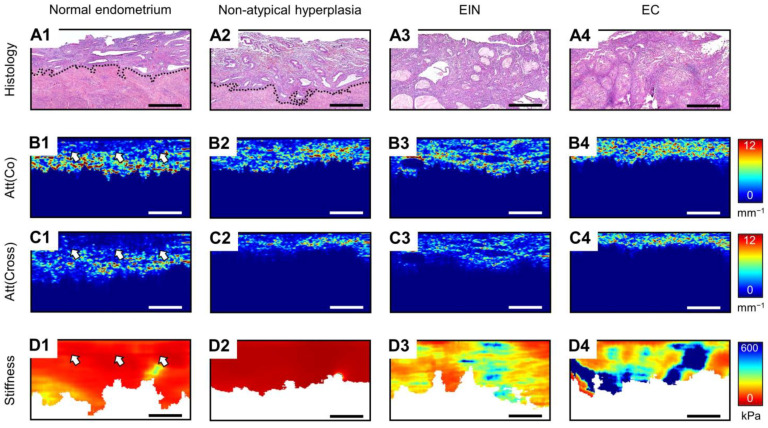
Comparison of the representative cases of normal endometrium (by example of secretory endometrium) (**A1**–**D1**), non-atypical endometrial hyperplasia (**A2**–**D2**), EIN (**A3**–**D3**) and EC (by example of low-grade EC) (**A4**–**D4**), where the following are presented: histological images (**A1**–**A4**), attenuation coefficient maps for co-polarization OCT images (**B1**–**B4**), attenuation coefficient maps for cross-polarization OCT images (**C1**–**C4**), C-OCE images (**D1**–**D4**); dotted lines and white arrows indicate border between endometrium and myometrium; bar size in all images is 0.5 mm.

**Figure A1:**
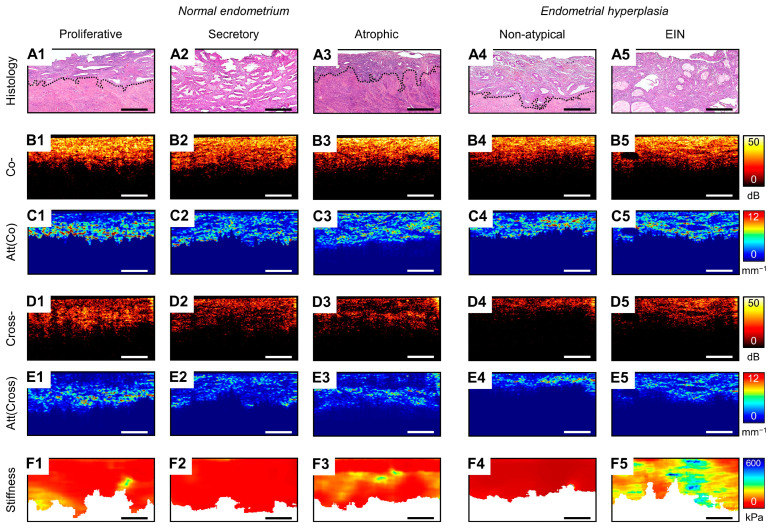
Representative cases of endometrial tissue in normal and with endometrial hyperplasia, where the following are presented: histological images (**A1**–**A5**), co-polarization OCT images (**B1**–**B5**), attenuation coefficient maps for co-polarization OCT images (**C1**–**C5**), cross-polarization OCT images (**D1**–**D5**), attenuation coefficient maps for cross-polarization OCT images (**E1**–**E5**), C-OCE images (**F1**–**F5**). Cases of normal endometrium include a study of premenopausal women with proliferative (**A1**–**F1**) and secretory (**A2**–**F2**) endometrium and postmenopausal women with atrophic (**A3**–**F3**) endometrium; endometrial hyperplasia tissues include non-atypical hyperplasia (**A4**–**F4**) and EIN (**A5**–**F5**); a dotted line in histological images represents a visible border between the endometrium and myometrium. The bar size in all images is 0.5 mm.

We conducted an initial examination of the normal endometrial tissue from premenopausal and postmenopausal women. Thus, cases of proliferative, secretory and atrophic endometria, characterized by different morphology, were analyzed. The proliferative endometrium consisted of uniformly and regularly distributed glands from round to tubular shape with numerous mitotic figures at glands and stroma in histological images (Figure 1(A1)). The visible border between the endometrium and myometrium is shown by a dotted line on histological images. The proliferating endometrium consisted of irregularly shaped, convoluted glands with nuclei located at the base of the cell and intraluminal secretion. The atrophic endometrium consisted of sparse glands with inactive low columnar or cuboidal cells and occasional cystic changes. They are characterized by their thinnest thickness. Representative histologic images of the normal endometrium of premenopausal and postmenopausal women are shown in Appendix A, Figure A1(A1–A3).

Qualitative comparison of OCT images in the co-/cross-polarization channels did not reveal differences in the intensity and depth of signal penetration for proliferative, secretory and atrophic endometrium (see Appendix A section, in Figure A1(B1–B5,D1–D5)). However, analysis of Att(co) values indicate a statistically significant difference of proliferative endometrium 1.73 [1.50; 1.99] mm^−1^ (Figure 1(B1)) from secretory 2.43 [2.16; 2.72] mm^−1^ and atrophic 2.96 [2.66; 3.26] mm^−1^ endometrium (*p* < 0.05) (Figure A1(C1–C3)), which correlated with the OCT study described in [32]. The measured CP OCT values are presented in Table 1. Analysis of Att(cross) values shows a statistically significant difference of proliferative 0.61 [0.47; 0.76] mm^−1^ (Figure 1(C1))/atrophic 0.75 [0.56; 1.01] mm^−1^ endometrium from secretory endometrium 1.37 [1.01; 1.65] mm^−1^ (*p* < 0.05) (Figure A1(E1–E3)). In addition, in the Att(co)/Att(cross) maps, the boundary between the endometrium and myometrium becomes clearly distinguishable due to better tissue contrast after image processing (boundary indicated by white arrows in Figure 1(B1,C1)). The capabilities of CP OCT in the identification of the boundary between the endometrium and myometrium are discussed in detail in Appendix A.

The novel C-OCE study results of normal endometrial tissue demonstrate low stiffness values of proliferative 60 [47; 73] kPa (Figure 1(D1)), secretory 51 [37; 66] kPa and atrophic 69 [31; 85] kPa endometrium. No statistically significant difference between the stiffness values of proliferative, secretory and atrophic endometrium was observed.

Next, we examined the endometrial tissue with non-atypical (benign) hyperplasia and EIN. They are much more likely to lead to malignancy [58,59]. In histological images, non-atypical hyperplasia is characterized by an abnormal proliferation of varying sized endometrial glands, leading to an increased gland-to-stroma ratio and thickening of the endometrium (Figure 1(A2)). EIN are characterized by the presence of glands with irregular morphology, in which pronounced cytological atypia can be observed (Figure 1(A3)).

**Figure 2:**
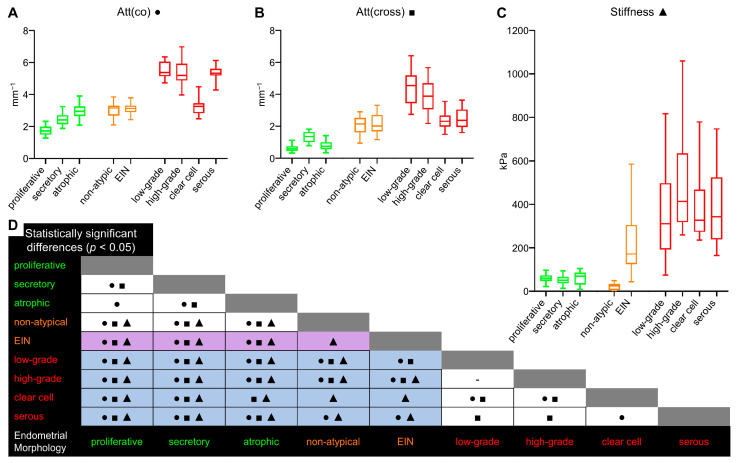
Boxplots for established Att(co) (**A**), Att(cross) (**B**), stiffness (**C**) values counted for each endometrial tissue types: normal endometrium, including proliferative, secretory and atrophy, indicated by green boxplots; endometrial hyperplasia, including non-atypical hyperplasia and EIN, indicated by orange boxplots; and different subtypes EC, including low-grade, high-grade, clear cell and serous, indicated by red boxplots. Centerline in the boxes—median; box limits—25th and 75th percentiles; whiskers—5th and 95th percentiles. Panel (**D**) shows the matrix of pairwise differential comparisons between different MM OCT parameters (●—Att(co) values, ■—Att(cross) values, ▲—stiffness values) for various endometrial tissue (Mann–Whitney U-test with a Bonferroni correction for multiple comparisons). The purple partitions indicate the possibility of differentiating EIN from benign endometrial tissues (non-atypical endometrial hyperplasia and normal endometrium). The blue partitions indicate the possibility of differentiating EC from endometrial hyperplasia and normal endometrium.

Qualitative comparison of OCT images in the co-/cross-polarization channels did not reveal any differences in the intensity and depth of signal penetration between non-atypical endometrial hyperplasia and EIN as well as between endometrial hyperplasia and the normal endometrium (Figure A1(B1–B5,D1–D5)). Some non-universal decrease in the depth of signal penetration of non-atypical endometrial hyperplasia (representative example on Figure A1(D4)) compared to normal endometrium and EIN was noted. Mapping, calculation and analysis of Att(co)/Att(cross) values also show the absence of differences between non-atypical endometrial hyperplasia 3.16 [2.69; 3.30] mm^−1^/2.15 [1.62; 2.53] mm^−1^ and EIN 3.14 [2.90; 3.32] mm^−1^/2.02 [1.69; 2.72] mm^−1^ (Figure 1(B2,B3,C2,C3)). However, we found a statistically significant difference in the Att(co) value between the endometrial hyperplasia and normal endometrium of premenopausal women (*p* < 0.05) and in the Att(cross) value between endometrial hyperplasia and normal endometrium of all women (*p* < 0.05) (Figure 2A,B). This indicates the greatest versatility of Att(cross), compared to Att(co), in diagnosing the endometrial hyperplasia of premenopausal and postmenopausal women. The more detailed statistically significant differences identified for different MM OCT parameters between morphological structures of normal endometrium and endometrial pathologies are presented in Figure 2D. It is worth noting the challenges associated with visual OCT diagnostics of EIN, which were also noted by other researchers. It operates with efficacy only in ~50% of cases [31]. Moreover, the difficulties of ultrasound/magnetic resonance diagnostics and the differentiation of physiological and pathological endometrial thickness in identifying endometrial hyperplasia/EC in premenopausal women are well known [60,61]. Thus, relative to ultrasound diagnostics of endometrial thickness and features for premenopausal women, analysis of Att(co)/Att(cross) values (by CP OCT) could be highly effective in identifying endometrial hyperplasia.

Compared with a normal endometrium, the C-OCE study of endometrial hyperplasia tissue established a decrease in stiffness values for non-atypical hyperplasia to 25 [6; 34] kPa and increase in stiffness values for EIN to 172 [124; 305] kPa (Figure 1(D2,D3). This once again highlights the malignancy potential of EIN, whose stiffness values are increased compared to non-atypical hyperplasia and a normal endometrium, and the same trends are observed for most types of human cancer [62,63]. In addition, statistically significant differences are established between non-atypical hyperplasia and EIN in tissue stiffness values (*p* < 0.05) (Figure 2C). Note that existing studies did not consider non-atypical (benign) hyperplasia at all in OCT diagnostics [32], the frequency of occurrence of which, as is known, prevails over EIN [64,65], and their optical properties are similar. This can be seen from our paper. Also, difficulties in diagnosing EIN are also observed when using hysteroscopy and/with transvaginal ultrasound diagnostics [66,67]. Nevertheless, the characteristic stiffness values of atypical hyperplasia/EIN statistically significantly differed from the normal endometrium (*p* < 0.05) (Figure 2D) demonstrating the scope of C-OCE (by stiffness values assess) for diagnosing endometrial hyperplasia and even identifying EIN.

**Figure A2:**
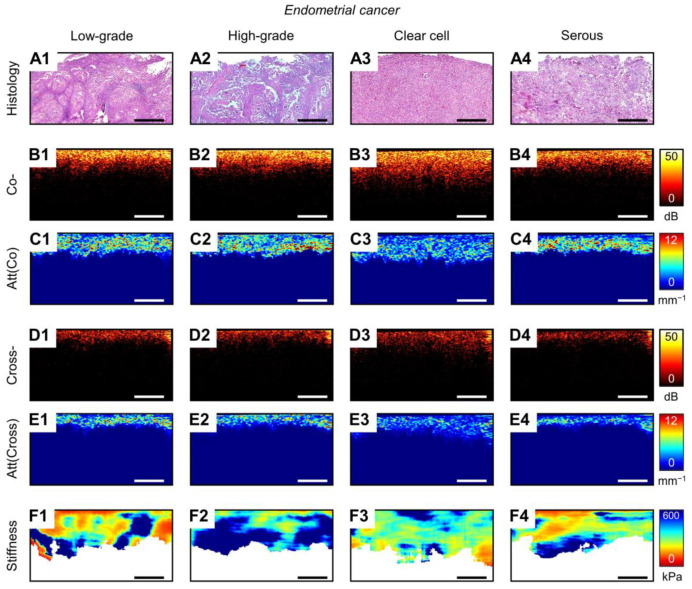
Representative cases of endometrial tissue with EC, where the following are presented: histological images (**A1**–**A4**), co-polarization OCT images (**B1**–**B4**), attenuation coefficient maps for co-polarization OCT images (**C1**–**C4**), cross-polarization OCT images (**D1**–**D4**), attenuation coefficient maps for cross-polarization OCT images (**E1**–**E4**), C-OCE images (**F1**–**F4**). Cases of EC include a study of next morphological subtypes: low-grade EC (**A1**–**F1**), high-grade EC (**A2**–**F2**), clear cell EC (**A3**–**F3**) and serous EC (**A4**–**F4**). The bar size in all images is 0.5 mm.

Finally, MM OCT and histological studies are performed on endometrial tissue samples with different morphological subtypes of EC, including low-grade, high-grade, clear cell and serous. In histological images, low-grade EC is characterized by confluent branching malignant glands with maze-like meandering interconnected lumens (Figure 1(A4)). High-grade EC are characterized by predominated solid growth with markedly altered cell cytology, often varying within a single tumor (Figure A2(A2)). Clear cell EC is characterized by papillary/solid architecture and scanty clear cytoplasm epithelial cells with pleomorphic nuclei (Figure A2(A3)), and serous EC is characterized by complex, branching papillae with a prominent tufting pattern and irregularly shaped glands lined by polygonal cells (Figure A2(A4)). These last two subtypes of EC are not associated with hyperplastic proliferation, and their statistics demonstrate a much poorer clinical prognosis [68,69].

A qualitative comparison of ECs by OCT images in the co-/cross-polarization channels revealed a reduction in the depth of signal penetration. This is a common characteristic of all EC morphological subtypes except for clear cell EC relative to a normal endometrium and endometrial hyperplasia (Figure A1(B1–B5,D1–D5) and Figure A2(B1–B4,D1–D4)). A similar result of a reduction in the penetration depth of the signal in EC correlates with other OCT studies of endometrial tissue [31,32]. The calculation and analysis of Att(co) values reveals statistically significant differences only between clear cell EC 3.25 [2.83; 3.46] mm^−1^ and other EC morphological subtypes (*p* < 0.05) (Figure 2A). In contrast, Att(co) values of other EC morphological subtypes, including low-grade 5.37 [5.14; 6.07] mm^−1^ (Figure 1(B4)), high-grade 5.20 [4.88; 5.93] mm^−1^ and serous 5.33 [5.19; 5.61] mm^−1^, were statistically significantly different from endometrial hyperplasia and normal endometrium (*p* < 0.05). The Att(cross) values of aggressive clear cell EC 2.32 [1.98; 2.68] mm^−1^ and serous EC 2.37 [1.95; 3.04] mm^−1^ are not statistically significant different from each other, but they are different from low-grade EC 4.56 [3.45; 5.19] mm^−1^ (Figure 1(C4)) or high-grade EC 3.88 [3.06; 4.70] mm^−1^ (*p* < 0.05), which are not different from each other (Figure 2B). Nevertheless, the Att(cross) values of serous EC and clear cell EC are not different from endometrial hyperplasia, whereas the Att(cross) values of low-grade EC and high-grade EC are statistically significant different from endometrial hyperplasia and normal endometrium (*p* < 0.05) (Figure 2D). The increase in Att(co) and Att(cross) values for EIN/EC compared to the normal endometrium could be due to the endometrial collagen formation and distribution features, which have been previously reported [70,71]. The obtained results from studying the polarization properties of EC by CP OCT imaging (by quantitative assess Att(co) and Att(cross) values) are indicative of the scope for differentiating most EC cases from endometrial hyperplasia and a normal endometrium.

The C-OCE study of EC tissue establishes a statistically significant increase in stiffness values to all EC morphological subtypes compared to normal endometrium/non-atypical endometrial hyperplasia (*p* < 0.05) (Figure 1(D1–D4)). However, we could not detect statistically significant differences in stiffness values between low-grade EC 311 [192; 497] kPa, high-grade EC 414 [318; 635] kPa, clear cell EC 327 [273; 468] kPa and serous EC 343 [239; 524] kPa (Figure 2C). We also could not find a statistically significant difference in stiffness values between low-grade EC and EIN (Figure 2D), but other EC morphological subtypes were statistically significantly different from EIN (*p* < 0.05). It is worth noting that difficulties in distinguishing EIN from EC are also observed during ultrasound diagnostics [65,66,67,72]. Despite this, we have established the prospects of C-OCE for identifying EC/EIN among benign and normal endometrial tissue.

In the next section, we will establish the most optimal diagnostic MM OCT criteria for identifying endometrial pathologies using CP OCT (by Att(co) and Att(cross) values) and C-OCE (by stiffness values). The calculation of diagnostic parameters for the established MM OCT criteria will determine the possible effectiveness and prospects for using MM OCT for diagnosing endometrial pathologies.

### 3.2. Diagnostics Parameters of MM OCT Criteria in EIN/EC Identification

Special attention is devoted to the diagnosis and therapy of EIN and EC in the current clinical practice of managing patients with corpus uteri pathologies [73]. Moreover, at the phase of primary clinical (non-pathological) diagnosis or monitoring of treatment efficacy of histologically diagnosed EC, the importance of identification/verification of the EC morphological subtype is insignificant [74]. Yet the need for developing a clinical tool for minimally invasive (low-traumatic) diagnosis of endometrial lesions is undiscussable [75]. Such a tool may be relevant, for example, in the primary diagnosis of focal lesions with unclear hysteroscopic features for the implementation of targeted biopsy and reducing the number of underestimations of EIN/EC [76,77] as well as for women diagnosed with EIN/early-stage EC receiving hormonal therapy and planning pregnancy after regression of pathology [78,79]. Therefore, in this section, we tried to distinguish the possible effectiveness of MM OCT in the diagnosis of clinically significant precancerous (EIN) and cancerous (EC) lesions versus benign endometrial tissues (non-atypical endometrial hyperplasia and the normal endometrium of premenopausal and postmenopausal women).

**Figure 3:**
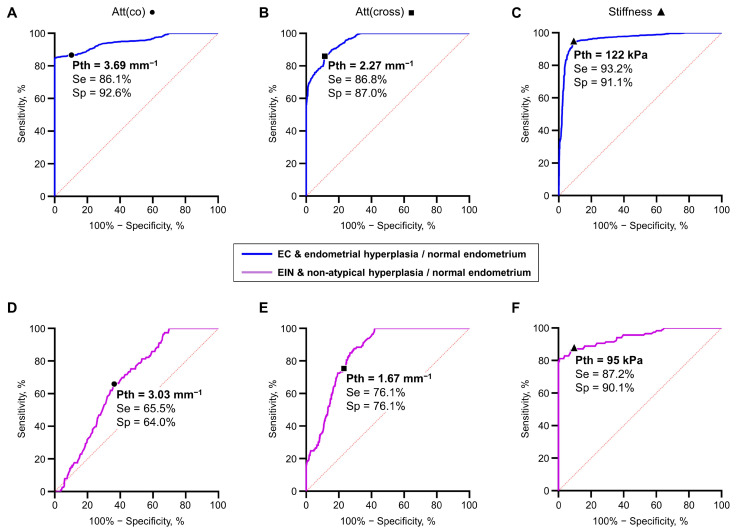
ROC curves showing the results for differentiation: EC from endometrial hyperplasia and normal endometrium (**A**–**C**), EIN from non-atypical hyperplasia and normal endometrium (**D**–**F**) based on the analysis of Att(co) (**A**,**D**), Att(cross) (**B**,**E**) and stiffness (**C**,**F**) values. Symbols (●—Att(co), ■—Att(cross), ▲—stiffness) on the curves represent choices of the threshold value (Pth) using a trade–off between the percentages of the false negative and false positive outcomes; designations: Se—sensitivity, Sp—specificity.

Figure 3A–C show ROC curves for the differentiation of EC (regardless of morphological subtypes) from non-tumorous endometrial tissue (endometrial hyperplasia and normal endometrium) using estimated values of MM OCT parameters: Att(co), Att(cross) and stiffness. The following threshold values were chosen for the MM OCT criteria: when using the Att(co) threshold value of 3.69 mm^−1^, the Sensitivity (Se) = 86.1% and the Specificity (Sp) = 92.6% (Figure 3A); when using the Att(cross) threshold value of 2.27 mm^−1^, the Se = 86.8% and Sp = 87.0% (Figure 3B); and when using the stiffness threshold value of 122 kPa, the Se = 93.2% and Sp = 91.1% (Figure 3C). The diagnostic parameters Se and Sp indicate high effectiveness and the potential of each MM OCT criteria in diagnosing EC. At the same time, the established diagnostic parameter of Se for hysteroscopic diagnostics EC is measured at about ~80% [80]. However, it is worth noting the recent progress in hysteroscopic diagnostics of EC, where the simultaneous use of artificial intelligence and a set of neural networks to improve the diagnostic accuracy of hysteroscopic diagnosis of clinically important endometrial pathologies made it possible to increase the values of diagnostic parameters to 86–91% [81,82].

A greater challenge is the instrumental clinical diagnosis of EIN, where, for example, the diagnostic parameter Se during hysteroscopy is measured at about ~75% [80,83]. To determine the possible effective of MM OCT criteria for diagnosing EIN, we plotted ROC curves for the differentiation of EIN from benign endometrial tissues (non-atypical endometrial hyperplasia and normal endometrium) (Figure 3D–F). The following threshold values were chosen for the MM OCT criteria: when using the Att(co) threshold value of 3.03 mm^−1^, the Se = 65.5% and the Sp = 64.0% (Figure 3D); when using the Att(cross) threshold value of 1.67 mm^−1^, the Se/Sp = 76.1% (Figure 3E); and when using the stiffness threshold value of 95 kPa, the Se = 87.2% and Sp = 90.1% (Figure 3F). These values of diagnostic parameters Se and Sp indicate the perspective possibilities and potential of tissue stiffness criterion (identify by C-OCE) in diagnosing EIN. Also, the established diagnostic parameters of C-OCE diagnostics of EIN significantly exceed the diagnostic capabilities of transvaginal ultrasound diagnostics in detecting EIN, which reach ~70% Se and Sp [72]. However, the use of artificial intelligence to improve the diagnostic capabilities of transvaginal ultrasound diagnostics of EIN allows increasing the Se and Sp values to ~85% [84]. It is also worth mentioning the progress in hysteroscopic diagnostics of EIN, where the use of computer-assisted tissue image analysis allows increasing the diagnostic parameters Se and Sp for detecting endometrial hyperplasia to ~80% [85]. At this point, the diagnostic parameters of the tissue stiffness criterion (identified in this study by C-OCE) established on the initial data set certainly require verification in a clinical format on a new test data set. Nevertheless, the tissue stiffness criterion looks more promising for addressing the challenges of high-precision low-traumatic diagnostics of precancerous/cancerous endometrial lesions, including EIN/EC.

It is also worth noting the difficulties of implementing OCT diagnostics in the human uterine cavity. The simultaneous need for contact of the OCT probe optical surface to the endometrial tissue and visual control/selection of a suspicious lesion area limit the adequacy of realizing OCT imaging of the endometrium to implementing it in one of the hysteroscope channels [30,86]. Here, we can note the progress in the development of intrauterine probes and the potential for their implementation for the diagnosis of human endometrial pathologies, where prototypes are already available for both OCT [31,32,87] and ultrasound/photoacoustic diagnostics [88,89]. Even more challenging is the implementation of C-OCE in the uterine cavity with the realization of a compression effect from the OCT probe on endometrial tissue. Some of the expected difficulties of such implementations and their solutions were described by us in a previous paper [44], where the main idea is the realization and adaptation of C-OCE palpation, which was previously shown by us [90] and other researchers [91,92].

Despite the established high values of diagnostic parameters for detecting EC (by CP OCT and C-OCE) and EIN (by C-OCE) performed on ex vivo endometrial tissue samples, further verification and possible correction of the selected threshold values of Att(co), Att(cross) and stiffness in larger-scale in vivo experiments is necessary. In addition, the simultaneous use of several OCT modalities for the detection of endometrial pathologies may significantly improve the values of diagnostic parameters in future clinical trials. Nevertheless, CP OCT and C-OCE modalities and the special processing of images (calculation and mapping of depth-resolved attenuation coefficient) used in this study for the first time for imaging human endometrial tissue demonstrate high efficiency and prospects for high-precision diagnostics of endometrial pathologies.

## 4. Conclusions

In summary, this study represents the first detailed investigation of the capabilities of MM OCT (CP OCT and C-OCE modalities) in detecting and differentiating endometrial tissue pathologies. Such multimodal imaging allowed us to qualitatively and quantitatively (by calculating the depth-resolved attenuation coefficient for CP OCT data and the stiffness values for C-OCE data) evaluate the polarization and elastic properties of endometrial tissue in norm and pathologies. Ex vivo surgical samples of normal endometrial tissue of premenopausal (in proliferative and secretory phases of the menstrual cycle) and postmenopausal (atrophic endometrium) women, as well as samples of endometrial tissue with hyperplastic changes (non-atypical hyperplasia and EIN) and EC different morphological types (low-grade, high-grade, clear cell and serous), were examined. The results of our extensive study demonstrate the features of changes in the polarization and elastic properties of endometrial tissue from norm to pathology and determine the trend changes by the measurement and analysis of MM OCT parameters (values of the depth-resolved attenuation coefficients Att(co) and Att(cross) and values of tissue stiffness). Analysis of the established differences in the values of MM OCT parameters allowed us to demonstrate the effectiveness of CP OCT/C-OCE in diagnosing the most clinically significant EIN/EC. The choice of threshold values for differentiating EC from endometrial hyperplasia/normal endometrium and EIN from non-atypical endometrial hyperplasia/normal endometrium and the subsequent calculation of diagnostic parameters revealed a high effectiveness of CP OCT (by Att(co) values = 3.69 mm^−1^, Se = 86.1%, Sp = 92,6%; by Att(cross) values = 2.27 mm^−1^, Se = 86.8%, Sp = 87.0%) and C-OCE (by stiffness values = 122 kPa, Se = 93.2%, Sp = 91.1%) in diagnosing EC as well as a high effectiveness of C-OCE (by stiffness values = 95 kPa, Se = 87.2%, Sp = 90.1%) in diagnosing EIN. The results and the developed diagnostic criteria to the best of our knowledge demonstrate for the first time the effectiveness of using MM OCT for diagnosing endometrial pathologies. The potential for the clinical implementation of such highly accurate and minimally invasive diagnostics can allow for a targeted biopsy of suspicious endometrial lesions and reduce the underestimation of EIN/EC as well as control the hormonal therapy effectiveness for EIN/early-stage EC in women planning pregnancy.

## Figures and Tables

**Table 1:** Results of calculation Att(co), Att(cross) and stiffness values of endometrial tissue in norm and pathologies.

Endometrial Morphology	MM OCT Parameters		
	Att(co), mm^−1^	Att(cross), mm^−1^	Stiffness, kPa
Normal endometrium			
Proliferative	1.73 [1.50; 1.99]	0.61 [0.47; 0.76]	60 [47; 73]
Secretory	2.43 [2.16; 2.72]	1.37 [1.01; 1.65]	51 [37; 66]
Atrophic	2.96 [2.66; 3.26]	0.75 [0.56; 1.01]	69 [31; 85]
Endometrial hyperplasia			
Non-atypical	3.16 [2.69; 3.30]	2.15 [1.62; 2.53]	25 [6; 34]
EIN	3.14 [2.90; 3.32]	2.02 [1.69; 2.72]	172 [124; 305]
EC			
Low-grade	5.37 [5.14; 6.07]	4.56 [3.45; 5.19]	311 [192; 497]
High-grade	5.20 [4.88; 5.93]	3.88 [3.06; 4.70]	414 [318; 635]
Clear cell	3.25 [2.83; 3.46]	2.32 [1.98; 2.68]	327 [273; 468]
Serous	5.33 [5.19; 5.61]	2.37 [1.95; 3.04]	343 [239; 524]

## Data Availability

The data presented in this study are available upon request from the corresponding author.

## Appendix A

This section presents representative MM OCT (CP OCT/C-OCE) and histological images of various endometrial conditions, including a normal endometrium of premenopausal (proliferative and secretory endometrium) and postmenopausal (atrophic endometrium) women (Figure A1(A1–A3,B1–B3,C1–C3,D1–D3,E1–E3,F1–F3)), endometrial hyperplasia (non-atypical hyperplasia and EIN) (Figure A1(A4,A5,B4,B5,C4,C5,D4,D5,E4,E5,F4,F5)) and different EC morphological subtypes (low-grade, high-grade, clear cell and serous) (Figure A2). Also, this section presents representative structural OCT images in co- (Figure A1(B1–B5) and Figure A2(B1–B4)) and cross-polarization (Figure A1(D1–D5) and Figure A2(D1–D4)) channels.

It is worth noting that the border between the endometrium and myometrium was almost indistinguishable in co-polarization OCT images of a normal endometrium (Figure A1(B1–B3)). Also, [31,87] report difficulties in identifying the border between the endometrium and myometrium by OCT. However, using the attenuation coefficient for co-polarization OCT images in some cases (where the growing endometrium is characterized by a small thickness), it is possible to establish the border between a statistically significantly different proliferative endometrium with low Att(co) values 1.73 [1.50; 1.99] mm^−1^ and myometrium with higher Att(co) values 3.06 [2.88; 5.23] mm^−1^ (*p* < 0.05) (Figure A1(C1)). At the same time, the thin atrophic endometrium did not differ in Att(co) values 2.96 [2.66; 3.26] mm^−1^ from myometrium, which did not allow establishing the border between them (Figure A1(C3)).

The OCT images of normal endometrial tissue in the cross-polarization channel demonstrate the possibility of visually detecting the boundary between a normal (proliferative/atrophic) endometrium, characterized by a lower signal intensity, and a myometrium, which is characterized by a higher signal intensity (Figure A1(D1,D3)). Att(cross) mapping demonstrated even more pronounced statistically significantly differences between a proliferative endometrium 0.61 [0.47; 0.76] mm^−1^/atrophic 0.75 [0.56; 1.01] mm^−1^ and myometrium 2.18 [1.67; 2.82] mm^−1^ (*p* < 0.05) (Figure A1(E1,E3)). In turn, the Att(cross) values of a secretory endometrium 1.37 [1.01; 1.65] mm^−1^ statistically significantly exceeded those of a proliferative/atrophic endometrium (*p* < 0.05), which confirms the capabilities of Att(cross) (and CP OCT in general) in differentiating proliferative and secretory endometria at different stages of the menstrual cycle (Figure A1(E1–E3)).

Additionally, it was possible to identify the border between the normal endometrium and myometrium with higher stiffness values 116 [59; 170] kPa, which were statistically significantly different from those of the proliferative 60 [47; 73] kPa/atrophic 69 [31; 85] kPa endometrium (*p* < 0.05) (Figure A1(F1,F3)), which was also shown by us in a preliminary study [93].
